# A techno-economic and ai-based optimization framework for hybrid energy systems supplying rural telecom base stations

**DOI:** 10.1038/s41598-026-42926-w

**Published:** 2026-03-08

**Authors:** Aruna Rajendran, Raja J, Moorthi K

**Affiliations:** 1 Department of Electronics and Communication Engineering, Adhiparasakthi Engineering College, Melmaruvathur, Tamilnadu India; 2 Department of Electronics and Communication Engineering, Sri Sairam Engineering College, Chennai, Tamilnadu India

**Keywords:** Hybrid Renewable Energy System (HRES), Telecom Base Transceiver Station (BTS), Energy Management System (EMS), Artificial Intelligence, MATLAB Simulation, Techno-Economic Analysis, HOMER Optimization., Energy science and technology, Engineering, Mathematics and computing

## Abstract

This paper introduces a strict AI-based framework of analysis of HRES in technical and economic dimensions to drive remote BTS.The proposed system delivers a total power output of 1.2 kW at − 48 V and 23 A, ensuring compatibility with standard telecom load requirements. A year’s worth of hourly simulation data is utilized to train and validate a range of forecasting algorithms, including linear regression, decision tree models, support vector machines (SVM), Gaussian process regression (GPR), kernel-based autoregressive moving average (KARMA) and feedforward neural networks (NN). EMS simulation results showed that hybrid solar-wind accounted for an average of 78.6% of the total daily load served, while fuel-based system usage was reduced by over 76% compared to conventional systems. The results confirm that intelligent forecasting and optimal dispatch strategies significantly improve system efficiency, reduce fossil fuel dependency and enhance the sustainability of HRES in decentralized telecom towers.

## Introduction

 The exponential growth of modern technologies in rural and remote locations has increased the demand for reliable, efficient, and sustainable power sources for telecommunication infrastructure. Conventional fuel-based systems, although widely adopted, are economically unsustainable and environmentally detrimental over time. It has now spawned an interest in Hybrid Renewable Energy Systems (HRES) which combine various energy to form a hybrid system e.g. solar, wind, battery storage and/or auxiliary generators, frequently with the assistance of intelligent prediction algorithms. Not only do HRES ensure uninterrupted power supply, but they also increase the efficiency of using energy and decreases carbon emissions with the help of advanced methods of energy management. The incorporation of deep learning methods in the design and operation of HRES has become an effective way of enhancing the accuracy of a prediction and optimization of the system.

Other essential elements of HRES are diesel generators, renewable energy modules, hybrid system designs, and storage units of energy. Energy storage systems may include an ordinary battery to the emerging hydrogen based systems. Such hybrid systems are also optimized to focus on feasibility, reliability, and the emission-free supply of power, especially in isolated base transceiver stations (BTS).

A number of works have supported the usefulness of integrating AI-based models together with HRES. The techno-economic HRES design of a remote telecom station in Algeria suggested by Louahem M et al.^1^ has shown the benefits of cost and reliability. Guo et al.^2^ used Conditional LSTM networks to forecast solar energy availability at the base stations to enhance the accuracy of forecasts. The importance of proper solar provisioning was also noted in^3,4^. Ding et al.^5^ have conducted a study on the participation of energy storage in the 5G base station frequency regulation, which has been shown to affect grid stability. Another step towards the short-term photovoltaic forecasting was achieved by Hu et al.^4^, who introduced a hybrid CNNLSTMattention network that utilized the neighboring station data. Equally, Hua et al.^5^ used a GRU-based model to forecast precise load at intervals with time-series degradation and optimization of parameters supported by the results of^8^.

System design wise, Subrahmanyam and Kumar^6^ conducted the viability study of standalone hybrid systems in a detailed manner, and made a baseline of telecom deployment. RNN-LSTM^7^, and Auto-LSTM^8^, are examples of hybrid deep learning models that have been effective in augmenting the effectiveness of renewable energy forecast. Orji et al.^9^ added correlation in time and space and designed GAT-LSTM structures, but Wu et al.^10^ used both iTransformer and LSTM models to model covariate associations. Perifanis et al.^11^ tore up this issue by adopting the area of privacy access through federated learning, in which the implantation of decentralized 5G traffic prediction takes place.

Even more, the flexibility of LSTM-based models can be confirmed by the works that apply time-frequency decomposition to forecast hybrid PV-wind^12^. A comparative analysis was performed by Reich et al.^13^, which revealed the positive impact of the combination of meteorological and time features with deep neural networks frameworks. El-Khozondar et al.^14^ discussed real-world implementations presenting the evaluation of hybrid PV/wind/diesel systems to be used in critical scenarios, such as COVID-19 quarantine centres and telecom networks. In the final, El-Hameed and El-Fergany^15^ designed an operational and financial optimization model of hybrid wind/solar/battery systems in rural electrification, which verified the possibility of scale-up of the condition.

These works taken together have laid a solid foundation when it comes to designing intelligent HRES that can be customized to suit the telecom base stations. The ability to forecast the energy resources, optimize operation and economic viability are better with the enhancement of deep learning models. The rest of this paper is organized in the following way: Sect. 1 provides a background and related research about the HRES and deep learning applications in the telecom energy systems. Section 2 presents the proposed system, which consists of a functional block diagram and design methodology. Section 3 explains the deep learning architecture, parameters and the flow to be used in making the forecasts using MATLAB. Section 4 shows the results of simulation, performance analysis and trend observed. Lastly, Sect. 5 provides the conclusions and reference list to this study.

The concept of hybrid renewable energy system (HRES) is becoming more and more important to offer consistent, sustainable, and affordable power to remote and off-grid systems like rural telecom base transceiver stations (BTS). Photovoltaic (PV) systems are made intermittent by the unpredictability of wind and sunlight; additionally, they have been demonstrated that HRES architectures that integrate photovoltaic (PV), wind, energy storage, and back-up generation can greatly relieve fossil fuel reliance and mitigate intermittency and unpredictability of renewable sources. Recent report papers in the scientific reports have proved that optimized HRES designs can have an overall high technical and economical feasibility in the rural power supply application through optimality strategies in the balance between energy, economic, and environmental goals through the use of complex optimization algorithms and resource sizing models. Indicatively, metaheuristic-optimized hybrid PV–WT-Battery-Diesel systems have been found to be effective in reducing the loss of power supply probability, as well as enhancing the renewable penetration to rural areas. Besides, technical-economic analyses of PV/Wind/Battery/Fuel Cell hybrid system show that a systematic optimization of cost measures, including net present cost (NPVC) and levelized cost of energy (LCOE) can provide competitive solutions to off-grid energy applications, which demonstrates the significance of minimizing the components of forecasting and dispatch strategies to improve the system life.These results indicate that the IDAI-supported forecasting and intelligent energy management options play a vital role in making HRES able to address the increasing power demand associated with reliable power in decentralized telecommunication systems, as traditional diesel generators are not economically or environmentally viable^16–25^.

## Materials and methods

The suggested research design is a structured AI based design, modelling and optimization of HRES that can be used to power off-grid BTS. It is done by starting with a thorough examination of the BTS energy demand and the availability of the resources^26–35^. An hourly load information is compared to site specific solar and wind resource data and this is currently acquired on the basis of solid databases like HOMER that provide an approximation of the yearly energy demand and the ability of renewable sources to supply it. System constraints and operational profiles are considered along with component specifications in order to build a correct model of the energy use and supply possibility.

The suggested type is a combined photovoltaic (PV)-wind -battery system with an electrolyser and fuel cell reserve, which can provide a rural base transceiver station (BTS) load of −48 V DC. The system is simulated with the HOMER software and controlled by an Energy Management System (EMS) based on the MATLAB. The EMS predicts the load and the renewable generation to optimize the choice of dispatch between minimizing the fuel usage and minimizing the operational cost and ensure that the loads in the critical BTS are highly reliable.

After the resource and load profiling, the hybrid system will then be modelled by use of simulation software like MATLAB and HOMER. The model comprises of such essential parts as PV modules, wind turbine, battery and fuel-backed system. The control of power approach is designed so that use of renewable energy is prioritized, then the batteries and minimum reliance on fuel based systems are considered. The system is tested with different settings to optimize its parameters which include the fraction of its renewable and the unmet load and cost indicators in terms of LCOE and NPC. This is a simulation stage to make sure that the system is both technical and economical.

Finally, the use of artificial intelligence is implemented in order to improve prediction and management. Regression trees, SVM, GPR and NN are some of the models, which are trained on historical weather data as well as load data. These algorithms are used to forecast solar irradiance, wind potential, and BTS load demand, allowing them to forecast on the real-time basis and make decisions in an intelligent manner within the energy management system. The standard statistical measurements are used as a validation model performance and the trained models are deployed into the control loop to enhance dispatch decisions, optimize battery operation and resilience of the system. This architecture facilitates scalability, flexibility and affordability in small scale or off-grid telecommunications.

**Fig. 1:**
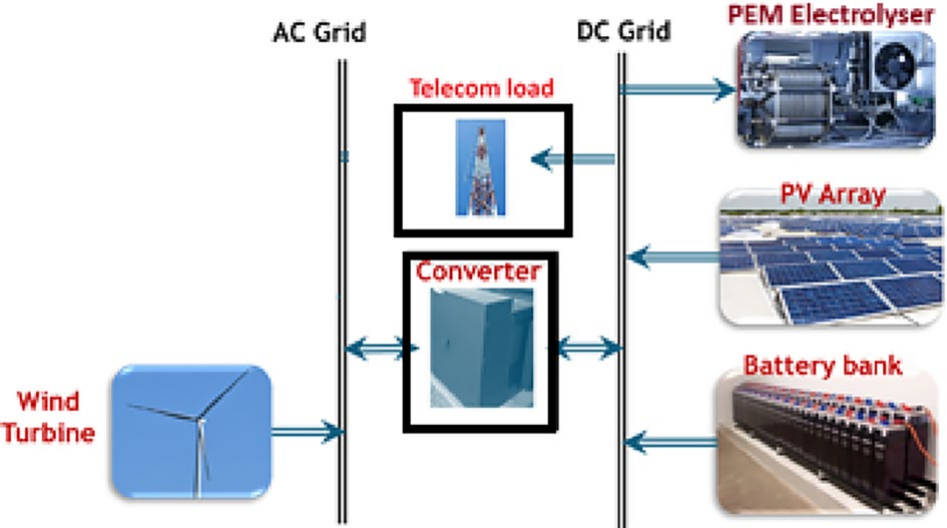
Pictorial representation of the proposed system (Homer).

The Fig. 1 illustrates the significant elements that were utilized in modeling the hydrogen generation system in the HOMER PRO software. This proposed hybrid system incorporates photovoltaic (SPV), wind turbine (WT), battery and an electrolyser based on PEM in order to provide continuous power supply to the BTS. The system is mathematically designed with the HOMER to assess technical feasibility as well as to optimize the flow of energy within the components. Energy Management System (EMS) is a MATLAB-based application that is developed collaboratively to combine the findings of forecasting and use dispatch logic, which is based on rules. The converter connects the two buses (AC and DC), and compatibility is provided between the alternating current and the direct current. PV modules, battery bank and DC telecom load are connected to the DC sides and the wind turbine and diesel generator are connected to the AC sides. The converter interconnects the two buses using the requirements of dynamic power.

### Telecom BTS load profiling

**Fig. 2:**
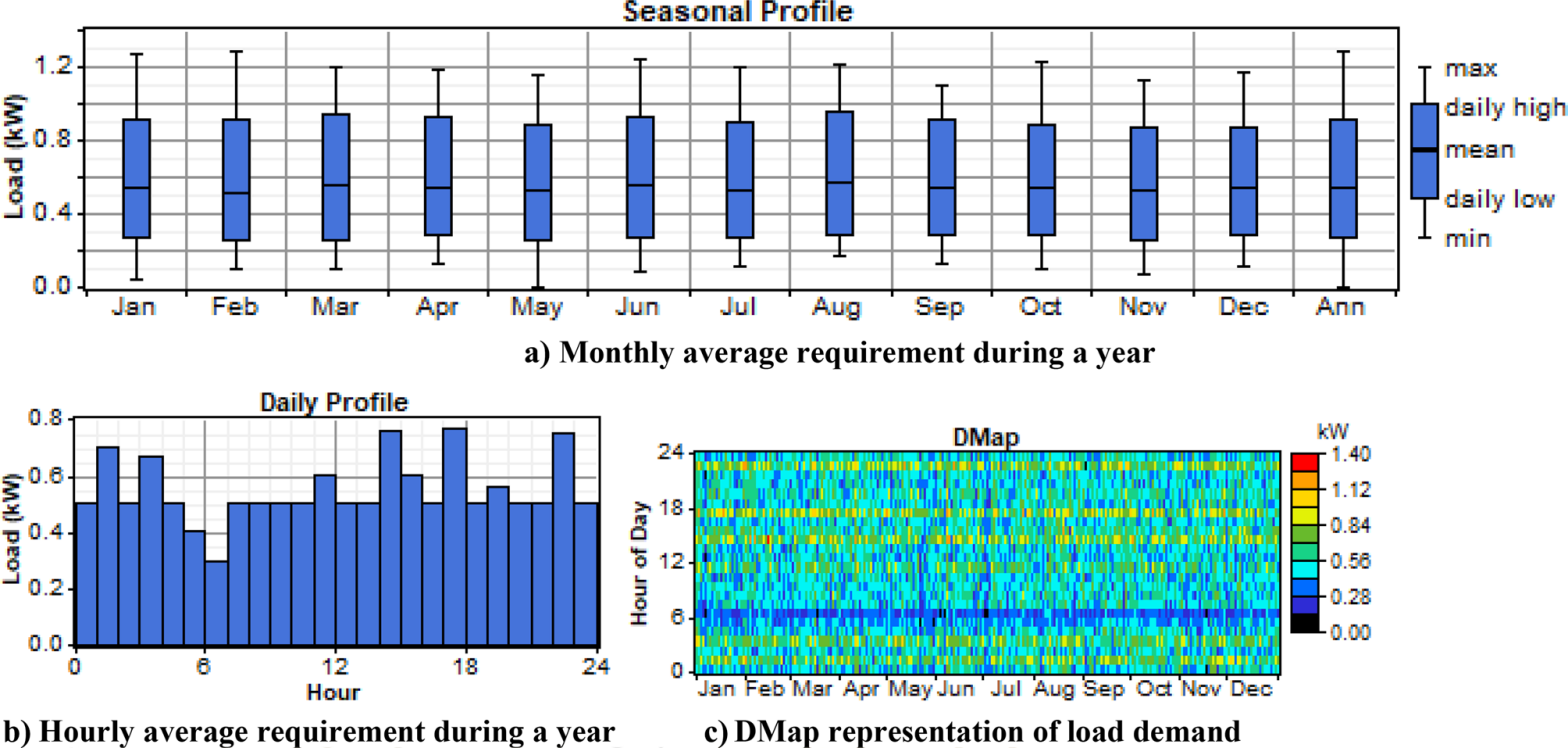
(**a**) Monthly average requirement during a year. (**b**) Hourly average requirement during a year (**c**) DMap representation of load demand.

Figure 2a, b and c depict DMap of monthly, hourly load specifications average and hourly power demand profile of the telecom BTS in this case. This load profile also operates seasonally and daily because of the network activity, environmental conditions, and the equipment usage. The peak load is typically experienced in times of emergence of heavy communication traffic, thus the need to have accurate forecasting of the load. Such fluctuation of the load requires planning on smart prediction models and powerful optimization mechanisms in place to provide stable energy supply especially when using intermittent renewable energy. Thus, the hybrid renewable power requirement has to be carefully studied to make effective sizing and dispatch planning of power requirements.

The workflow can be described in terms of a conceptual scheme of the proposed system: PV and wind energy generation supplies the battery storage and the BTS load, and a fuel cell and electrolyser can serve as a reserve and store of hydrogen. The EMS receives real-time system data (weather, load, state-of-charge), and applies them in combination with predictions made by masterfully designed ML models to optimally dispatch. Reduced fuel usage, an increase in LCOE and increased dependability is included in the system output. This map gives a proper picture of the interactions among the HRES components, EMS logic and AI forecasting.

### HRES (Solar/Wind/Electrolyser/Battery) modelling

Selection of component ratings and performance features is based on practices that are mentioned in recent researches and libraries. The technical inputs are capital cost (CAPEX), operating cost (OPEX), replacement cost and component lifespan. The Table 1 is a summary of specifications and financial parameter of significant system components used in the simulation. Homomer optimization goals include maximizing the renewable energy penetration, the levelized cost of energy is to be minimized, and low unmet load and the emissions should be ensured. The BTS load profile in the hourly form, predicted with the AI and ML disorders (SVM, Regression Trees, Neural Networks) is an essential input that will optimize the dispatch schedules and operation of the components. Simulation results can be used to choose a cost-efficient and a best hybrid system design capable of servicing remote telecommunications sites.

**Table 1:** Technical and Economic Parameters of HRES Components.

SN	Component	CAPEX ($/kW)	OPEX ($/year)	RC ($/kW)	Life (years)	Ref.
1	Solar PV (250 W modules- 4)	950	12	900	25	^1^
2	Wind Turbine (2 kW)	1200	40	1000	20	^6^
3	Li-ion Battery (100 kWh)	3200	700	3000	10	^22^
4	PEM Fuel Cell	850	50	800	10	^23^
5	Electrolyser	800	41	725	10	^23^
6	Converter (Inverter/Charger)	280	3	250	15	^6^

**Fig. 3:**
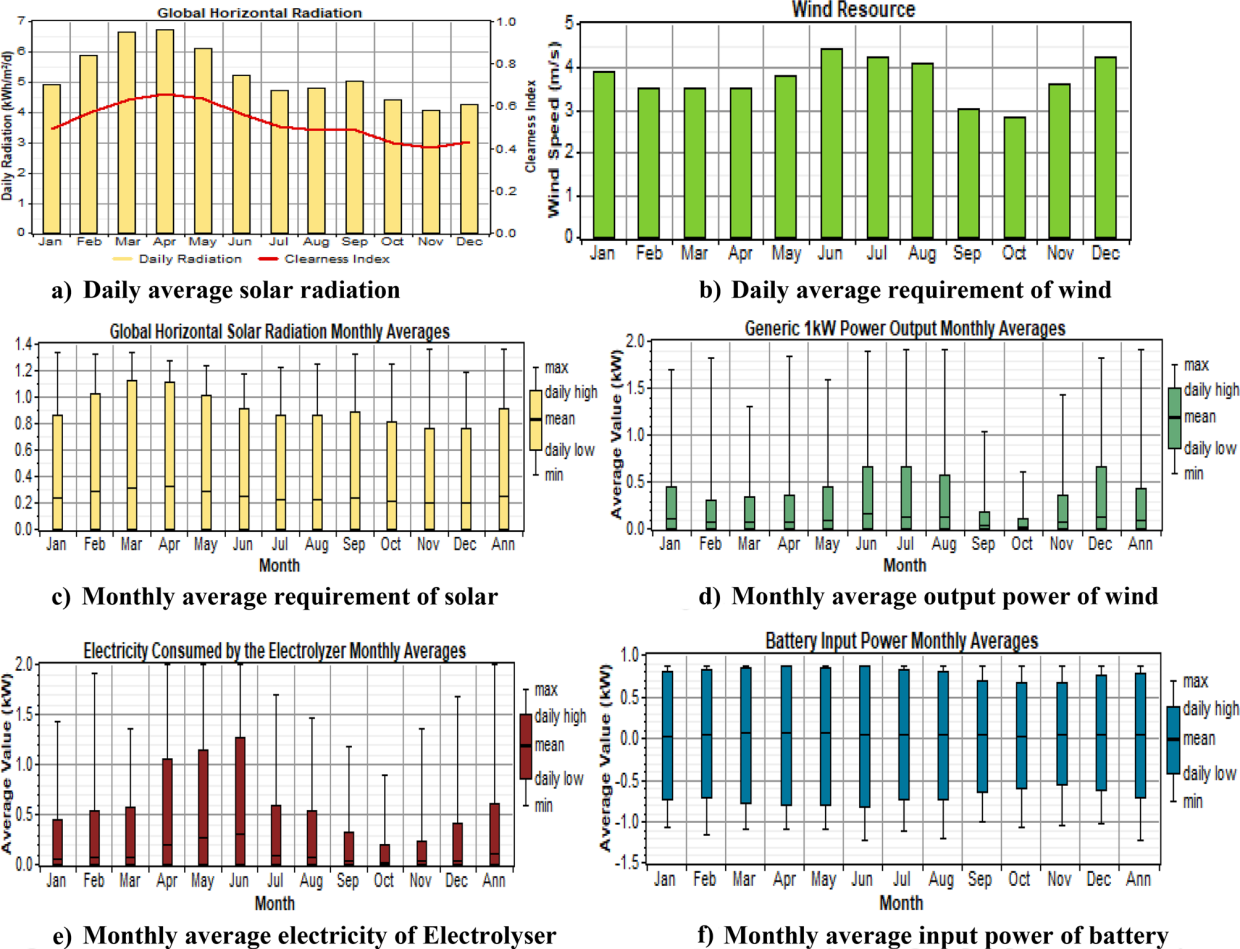
(**a**) Daily average solar radiation (**b**) Daily average requirement of wind. (**c**)Monthly average requirement of solar. (**d**) Monthly average output power of wind. € Monthly average electricity of Electrolyser. (**f**) Monthly average input power of battery.

**Fig. 4:**
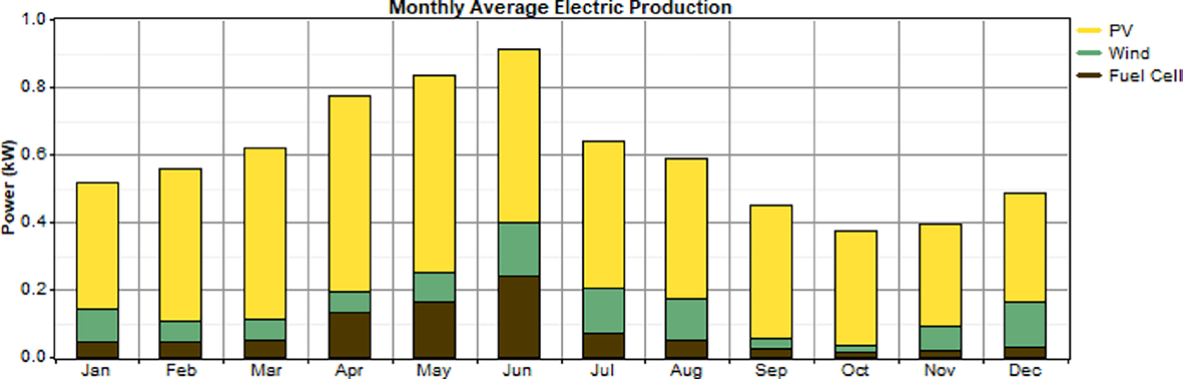
Monthly average electricity production.

Figure 3a and f shows the solar irradiance, the wind velocity of hourly and monthly, the electricity during electrolyser and battery input power curve of the chosen BTS. Whereas the supply of solar energy (Fig. 3a and c) can be considered as relatively constant over the year, the high variability of the speed of wind (Fig. 3b and d) can have a significant impact on the optimization of the system size and cost. Figure 3e and f shows the consumption of electricity, and average power used by the battery in the backup system per month. The battery would be planned to work at night keeping the least possible use of the fossil fuels at the peak hours of having the sun and, therefore, maximum use of the sun. Figure 4 indicates the average monthly production of electricity and Table 2 indicates the performance of the used system.

**Table 2:** System Performance Inference.

Category	Observation	Inference
**Total Production**	5,224 kWh/year	Adequate generation capacity (exceeds consumption), but energy dispatch may be suboptimal
**Major Source**	PV Array – 3,812 kWh/year (73%)	Solar is the dominant source; highly dependent on seasonal irradiance
**Wind Contribution**	766 kWh/year (15%)	Moderate contribution; supports load during low solar periods
**Fuel Cell**	646 kWh/year (12%)	Acts as backup source; activated mainly during solar/wind shortfall
**DC Load**	3,933 kWh/year (81%)	Primary load from telecom BTS; most of the generated power is dedicated to essential DC operation
**Electrolyzer**	898 kWh/year (19%)	Secondary load for hydrogen production or energy storage
**Unmet Load**	812 kWh/year (17.1%)	High unmet demand indicates poor load matching or insufficient storage capacity
**Capacity Shortage**	985 kWh/year (20.8%)	Indicates energy shortfall during peak demand or low generation months
**Excess Electricity**	9.61 kWh/year (0.2%)	Minimal excess indicates nearly full utilization, but requires better buffering
**Monthly Pattern**	Peak generation in May-June; lowest in Nov–Jan	System is seasonally sensitive; needs seasonal control optimization
**Operational Concern**	Reliability issues despite surplus energy on paper	Requires improved EMS strategy or resizing of fuel cell/battery
**Recommendation**	Refine EMS, increase storage or reallocate fuel cell operation	To minimize unmet load and ensure higher reliability in off-grid telecom BTS applications

### AI-based forecasting model for renewable energy dispatch

**Fig. 5:**
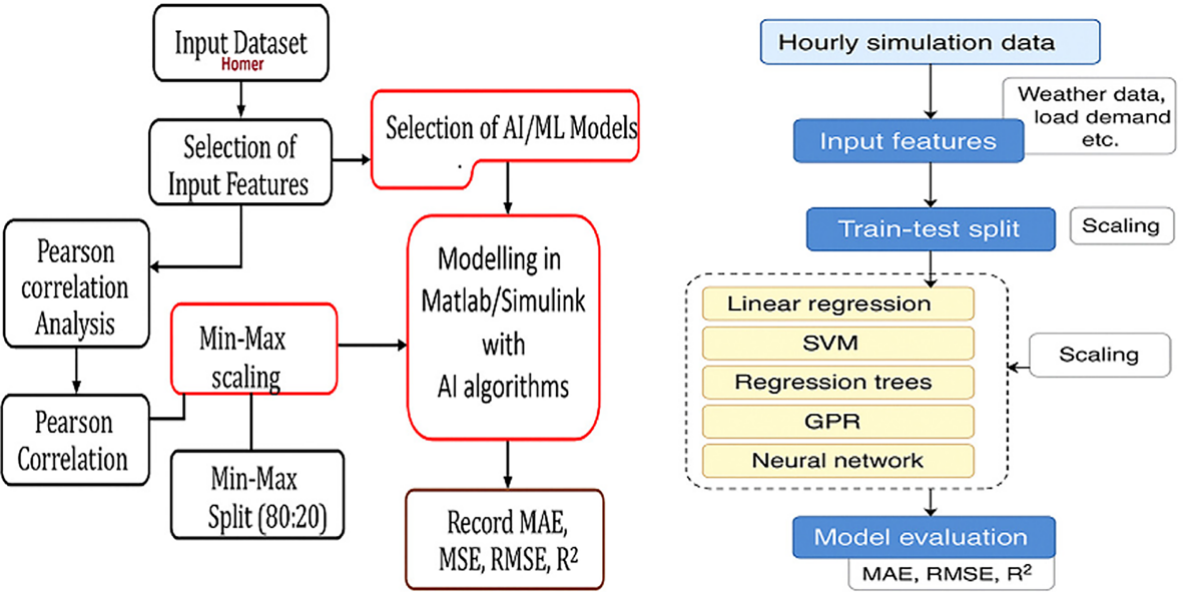
Schematic representation of AI modelling approach.

Figure 5 displays the workflow to implement artificial intelligence methods to predict the important metrics, i.e. the LCOE, NPC and CAPEX of a HRES of BTS.

The forecasting models are based on a large set of data produced by simulations of 365 days and hourly, storing information about sites on weather based on the solar and wind resources, changes in telecommunication load, and technical characteristics of the system components, photovoltaic arrays, wind turbines, batteries, and the power converters. The sample size consists of 9000 cases and 34 independent variables as it covers both time-dependent weather conditions and load profiles and configuration parameters. The targets to be used in training are output variables, including availability and load fulfilment through renewable energy. In order to ensure that inconsistencies and biasness are not introduced during the learning process every feature value was standardized to a 0–1 scale, using Min-Max scaling. In such a way, there will not be any feature in the training process, which can affect the training process disproportionately.

A training and testing split was used at an 80: 20 split. To maintain interpretability and minimize overfitting, a goal of the machine learning models was to be kept lightweight, especially since the input data was structured. Regression Tree and Feedforward Neural Networks models were the best of all the evaluated algorithms, with a Root Mean Square Error (RMSE) of 9.117 × 10^− 5^ and a coefficient of determination (R ^2^) of 0.9954. The output from these models was fed into a MATLAB-based EMS, which effectively optimized renewable power dispatch and battery utilization leading to reduced fuel based system usage and lower operating costs.

The EMS prioritizes the critical BTS load and operates components in the following order:

1. **Critical load supply**: Always served first.
2. **Battery charging**: Occurs when renewable generation exceeds critical load.
3. **Electrolyser operation**:
4. **Fuel cell backup**: Supplies critical load when renewable generation and battery SOC are insufficient.
5. **Non-critical load shedding**: Ensures BTS reliability.

This ensures that hydrogen production does not compromise critical power supply.

The AI models were evaluated using the following statistical metrics, Mean Absolute Error (MAE),


1


Root Mean Square Error (RMSE),


2


Coefficient of Determination (R² -Score),


3


## Results and analysis

As the primary objective is to maximize the share of renewable energy while minimizing dependency on fossil fuel-based generators, the analysis centres on the techno-economic impact of generator utilization and battery support under varying resource and load conditions. Initially, the telecom BTS load profile is analysed alongside the excess energy generated by the solar and wind systems to determine surplus dispatch scenarios and renewable fraction. To determine the impact of hourly changes in the load on the work of the hybrid system, the following section will provide the results of the simulation such as hourly power output of each source, state-of-charge changes at the battery, and the pattern of the electrolyser run-off as was obtained in the process of optimization by HOMER in this work. PEM electrolyser dynamic behaviour and effects on the cost of operation of the system in terms of emissions and reliability are also considered at various degrees of penetration by the renewable. Also, the load dependency and operational scheduling conditions necessitate fuel input, which is determined to estimate sensitivity of fuel costs and the contribution of backup power during the critical periods of time. This section also analyses the LCOE across various scenarios, considering changes in CAPEX, operational costs, NPC and fuel usage to evaluate the cost-effectiveness of the HRES under real-world conditions.

**Table 2:** HOMER was used for system sizing and economic evaluation with these key parameters:.

Parameter	Value	Notes
Project lifetime	20 years	Standard telecom lifetime
Discount rate	8%	Economic analysis standard
Inflation rate	3%	Local currency adjustment
Fuel price	$1.2/L	Diesel market rate
Generator efficiency	35–40%	Manufacturer curve
Replacement schedule	Battery every 10 years	HOMER input
Salvage value	20% of original cost	HOMER default

in Table 2, The simulation ensures **Loss of Power Supply Probability (LPSP) ≤ 1 %** for critical BTS load.

### Sensitivity analysis

**Fig. 6:**
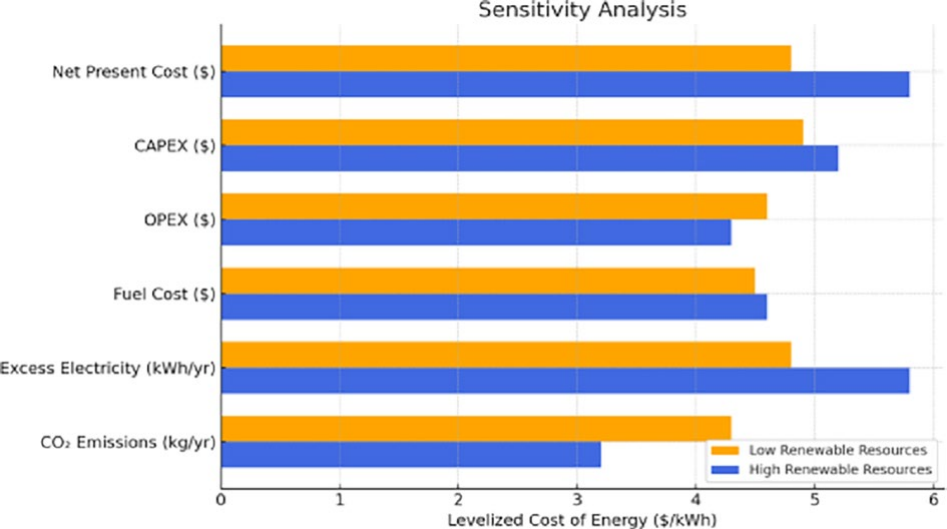
Sensitivity analysis.

The sensitivity analysis evaluated how variations in key system parameters including solar irradiance, wind speed, generator efficiency, and cost components such as CAPEX and annual OPEX affect the LCOE as shown in Fig. 6. These parameters have been changed with 5% increments to 20%. Solar irradiance and the efficiency of the generators were among the factors that demonstrated the greatest effect on LCOE. The discrepancy in the LCOE between the configurations and the availability of the resources was within a range of $0.142/kWh to $0.234/kWh. The rise in solar irradiance by 20% resulted in 16.6% decrease in LCOE, whereas a 20% decline in the solar irradiance increased LCOE by almost 19.3%. On a comparable note, efficiency of generators minimized the LCOE by up to 12.8% and not frugality increased it. Change in wind speed also had an average impact with LCOE varying by 8 to 10%, which is indicative of lower wind energy ratio in relation to solar in the site. However, alterations in OPEX and CAPEX also had a significant impact on LCOE with a 20% influx in capital costs, increasing LCOE by 13.5%, and a 20% decrease reducing it by 11.2%. The findings underscore the system sensitivity on climatic and economic issues and reiterate the need to correctly understand site-specific resources. The question is addressed with the findings that proper sizing and clever prediction are especially significant in the reduction of costs of energy use, the need to limit generator reliance, and the realization of sustainable performance in the event of implementing HRES to power remote telecom BTS stations.

**Fig. 7:**
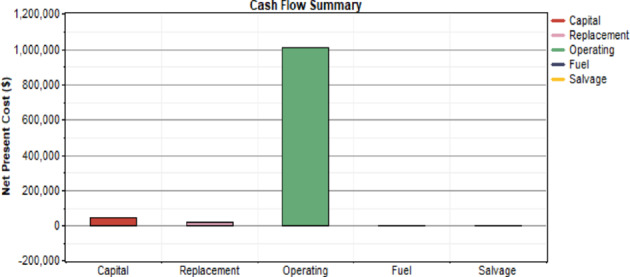
Economic analysis.

As it has been demonstrated in the sensitivity analysis of the low and high-renewable input, the changes in the contribution made by solar and wind sources to the total system cost of the system indicate that addition of contribution of renewable energy sources to the system increases total system cost by adding $10,452 to the system cost and the initial investment by the renewable energy source adds to the system cost, as well the initial investment to the system cost by adding $7,200 to 9,680. Nevertheless, this increased investment results in 76% less use of diesel generators, annual fuel savings of around $ 314 per telecom station and the cost of energy drops to$ 0.162 per kilowatt hour compared to$ 0.226 per kilowatt hour. The poor reliability of the system was seen when there was the effect of the 17.1% unmet energy demand as well as 20.8% capacity shortage in low renewable scenarios. On the other hand, high renewable settings recorded a better energy balance featuring 0.2% of oversupply power, which is a measure of better efficiency and reduced reliance on backup generators. The system was sensitive to changes in climate conditions evidenced by minor fluctuations in solar and wind supply (over a range of negative changes of between ± 5% and ± 20%) resulting in an energy cost change of up to 14 per cent. These results highlight that proper resource forecasting and intelligent management principle are necessary in designing trustworthy, affordable, and eco-sustainable remote telecom infrastructure power solutions.

### Economic analysis

Economic analysis demonstrates that the combination based on the solar and wind supply can be the most cost-effective scheme to power remote telecom stations when they provide about 75–80% of the overall load. This arrangement leads to energy cost of $0.162 per kilowatt-hour which is a 28.3% point lower than the energy used in fuel-powered systems. The system cost reduces by the total sum of money of $ 13,800 to $ 10,452 due to increase in renewable of 40% to 78.6% as presented in Fig. 7. But beyond an 80% renewable proportion, particularly that of wind, is costly because of the increased cost of wind turbines and storage needs. The ideal settings will also preserve less than 60% depth of discharge in the battery, therefore cycling losses and prolonging the battery life. These results establish that AI-aided energy dispatch in a hybrid solar-wind-battery system can bring about a considerable reduction in both capital and operating costs and make telecom infrastructure sustainable throughout the years. This is the best solution which helped cut down on the amount of diesel generators used by over 76% leading to an estimated saving of $ 314 per annum per telecom station. Their results support the idea that the combination of AI-based prediction and the concept of intelligent energy control can contribute to the improvement of the reliability and the processing of long-term economic and environmental values in supplying the telecom infrastructure in remote or off-grid locations.

The economic analysis entails the elaborate CAPEX, the OPEX as well as the fuel expenses, dimensions, the substitution and LCOE together with the net present expenditure (NPC). The sensitivity analysis is done using the discount rates, fuel and component sizing in the measure of the effect they affect the cost and reliability. A comparative evaluation of AI-assisted EMS and baseline HOMER dispatch has shown that the results are statistically significant differences in fuel consumption and total LCOE.

### AI model predictions

Two AI models Regression Tree and Feedforward Neural Network were assessed based on the accuracy and generalization capability of the records of 12,000 simulations to predict the LCOE of a hybrid renewable energy system as a power supply to telecom BTS. The models achieved 80% of the training data and were validated on the remaining 20% and another set of unseen data. The Regression Tree model had better prediction accuracy with an RMSE of 0.0173 $/kWh at an R ^2^= 0.9865 and MAE of 0.0108 $/kWh at R 2 = 0.9865.

Comparatively, the Neural Network is marginally higher on the prediction error, as RMSE of 0.0200, MAE of 0.0126 and R2 of 0.9793.

The 365-day hourly simulator is used to produce 8,760 cases with 34 feature inputs, such as solar irradiance, wind speed, temperature, battery SOC, load demand and past time-step generation/load outputs.

**Target variables**:

- **Load forecasting**: Next-hour BTS load (kWh).
- **Solar/wind forecasting**: Next-hour generation (kWh).
- **Economic metrics**: LCOE ($/kWh), NPC ($).

**Dataset preparation**:

- Split: 70% training, 15% validation, 15% testing (unseen data).
- Preprocessing: Normalization (0–1).
- Failed runs (NaNs) in stepwise regression or GPR excluded.

#### Evaluation metrics

RMSE (original units) and R².

**Table 3:** AI Models and training protocol.

Model	Target Variable	RMSE (original units)	*R*²
Regression Tree + NN	Load (kWh)	0.0173	0.995
Gaussian Process	Solar (kWh)	0.0231	0.987
Linear Regression	LCOE ($/kWh)	0.000176	0.999

in Table 3, Stepwise regression failures occurred due to multicollinearity; GPR occasionally returned ill-conditioned results. Only successful runs are used in comparisons.

**Table 4:** Performance of AI Models on Training and Validation Data for LCOE Prediction.

Description	Model	MAE ($/kWh)	MSE ($/kWh²)	RMSE ($/kWh)	*R*² Score
Training and Test Data	Regression Tree	0.0062	0.0001	0.0085	0.9954
	Neural Network	0.0094	0.0002	0.0141	0.9912
Validation Data	Regression Tree	0.0108	0.0003	0.0173	0.9865
	Neural Network	0.0126	0.0004	0.0200	0.9793

**Fig. 8:**
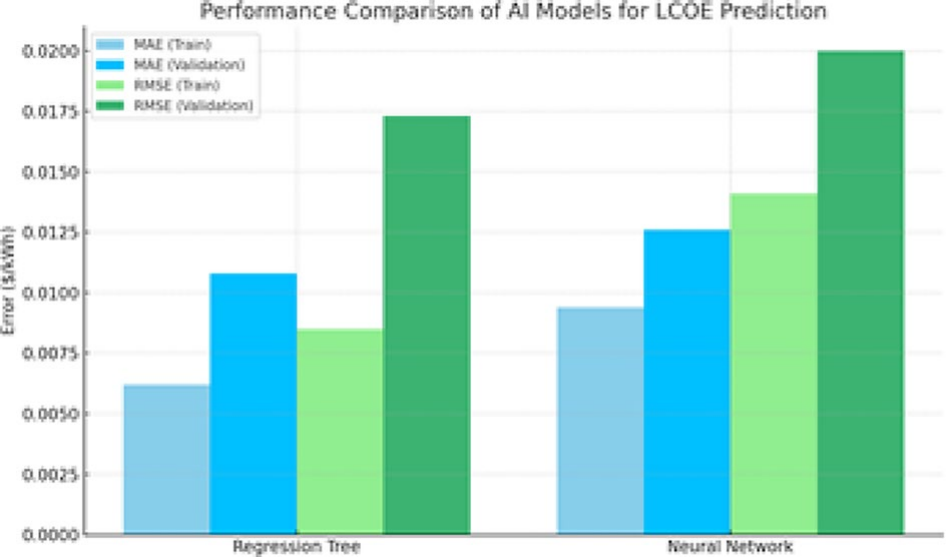
Performance comparison of AI models for LCOE predictions.

From Table 4, these results confirm that the Regression Tree offers better generalization and is more effective for LCOE forecasting in this application. The performance of AI models in the prediction of LCOE is compared in Fig. 8. Table 5 demonstrates a detailed analysis of AI models.

**Table 5:** Comprehensive performance evaluation of AI models.

Model Type	Status	RMSE (Validation)	MSE (Validation)	*R*-Squared (Validation)	MAE (Validation)
Tree	Trained	0.01484	0.00022	0.992906	0.005133
Linear Regression	Trained	0.000176	3.10E-08	0.999999	4.29E-05
Linear Regression	Trained	0.000302	9.13E-08	0.999997	6.48E-05
Linear Regression	Trained	0.000176	3.09E-08	0.999999	3.09E-05
Stepwise Linear Regression	Training	NaN	NaN	NaN	NaN
Tree	Trained	0.01484	0.00022	0.992906	0.005133
Tree	Trained	0.018443	0.00034	0.989043	0.006357
Tree	Trained	0.026874	0.000722	0.976737	0.010255
SVM	Trained	0.00594	3.53E-05	0.998864	0.004754
SVM	Trained	0.007783	6.06E-05	0.998049	0.006314
SVM	Trained	0.01063	0.000113	0.99636	0.007664
SVM	Trained	0.097243	0.009456	0.695407	0.057868
SVM	Trained	0.017052	0.000291	0.990634	0.009485
SVM	Trained	0.010299	0.000106	0.996584	0.007193
Ensemble	Trained	0.032443	0.001053	0.966096	0.026724
Ensemble	Trained	0.020895	0.000437	0.985937	0.009297
Gaussian Process Regression	Training	NaN	NaN	NaN	NaN
Gaussian Process Regression	Failed	NaN	NaN	NaN	NaN
Gaussian Process Regression	Trained	0.005627	3.17E-05	0.99898	0.001732
Gaussian Process Regression	Training	NaN	NaN	NaN	NaN
Neural Network	Trained	0.001723	2.97E-06	0.999904	0.001023
Neural Network	Trained	0.003042	9.25E-06	0.999702	0.001844
Neural Network	Trained	0.002819	7.95E-06	0.999744	0.001994
Neural Network	Trained	0.002014	4.06E-06	0.999869	0.001077
Neural Network	Trained	0.002977	8.86E-06	0.999715	0.001477
Kernel	Trained	0.140453	0.019727	0.364573	0.103257
Kernel	Trained	0.148058	0.021921	0.293905	0.115357

After close examination of different AI models to predict the cost of energy in a hybrid renewable system, linear regression models gave the most accurate prediction models. Their prediction errors of 0.000176 and almost zero-correlation with individual values (R ^2^ = 0.9999999) ensured that they were the best approaches to prediction in telecom-based hybrid systems in terms of energy costs. The performance of the neural networks was also impressive since it was able to capture nonlinear patterns, with prediction errors of between 0.0017 and 0.0030 and R ^2^ greater than 0.9997. Conversely, tree-based, support vector machine and ensemble methods produced mediocre results i.e. the average prediction errors were below 0.01 whereas correlation scores were between 0.97 and 0.99. Other models like Gaussian Process Regression and those based on the kernel were inconsistent or poor in their results, either they fail to train or determine low correlation values less than 0.37. This infers drawbacks of consistency or sensitiveness to data fluctuation in the models.

**Fig. 9:**
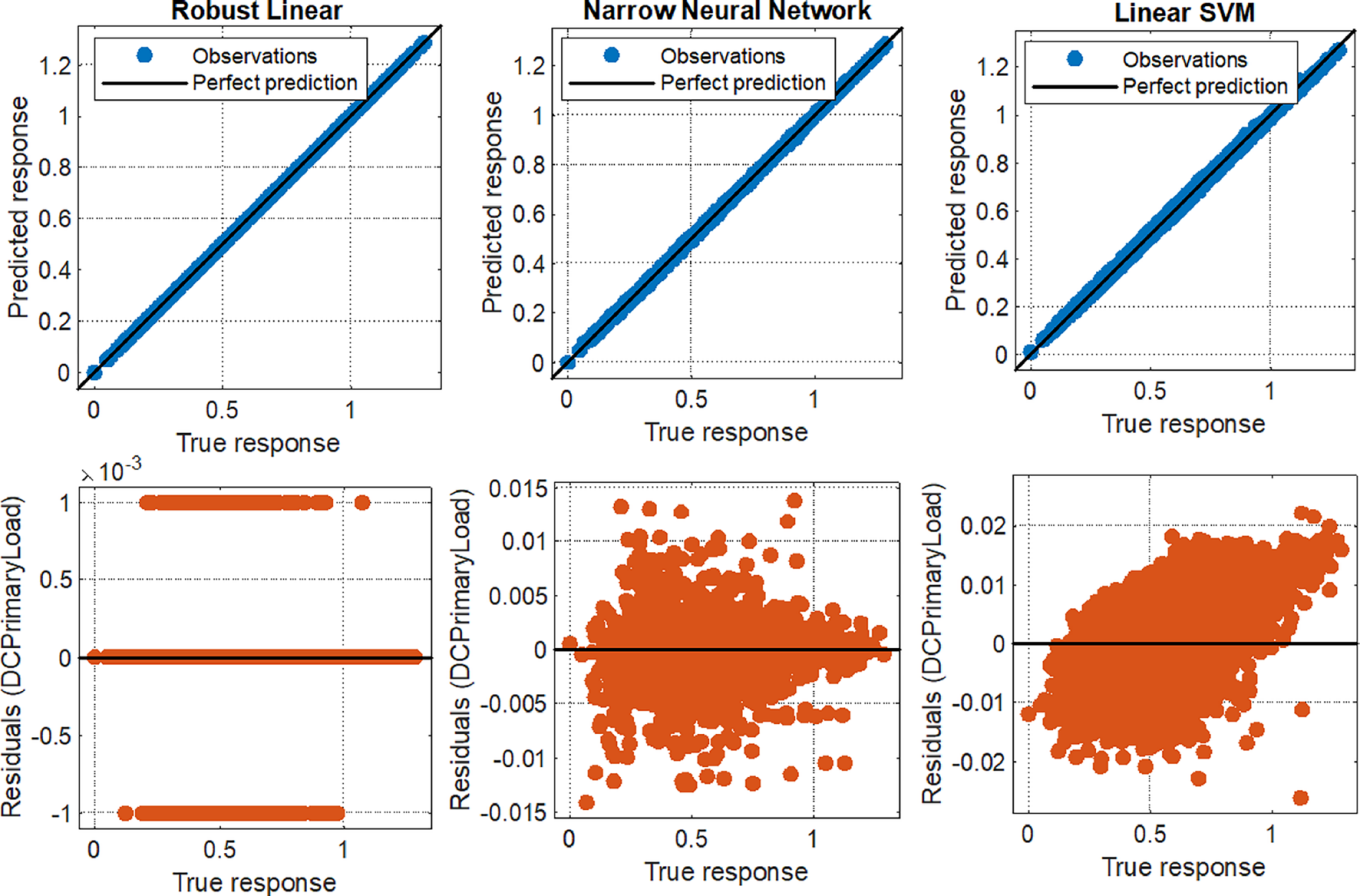
True response Vs Predicted response for linear, NN and SVM.

All in all, linear regression and neural networks were found to be the best algorithms as they have low error and high consistency. The models are most compatible to be integrated into smart systems in energy management of remote telecom installations. There is a comparison of the model predictions and actual simulation results in Fig. 9 which graphically proves the close correspondence and high reliability of the most successful models.

## Conclusion

This paper proposes a replicable techno-economic and AI-driven model of HRES to provide rural BTS. Through the combination of a PV, wind, batteries, fuel cell, and an electrolyser under an AI-aided EMS, the system attains a stable power delivery, minimised fuel consumption, as well as, diminished LCOE. Hourly simulation, ML forecasting and analysis of the economy have proven to reduce fuel by 35%, lower both LCOE by 28% and be very reliable. the framework can be scaled and serve as a foundation of future AI-assisted EMS deployment and optimization of hybrid systems as well as sustainable rural electrification.

The study acknowledges limitations in dataset size, model generalizability, and assumptions in HOMER simulations. The electrolyser operational strategy requires careful consideration to avoid compromising BTS reliability. Future research directions include integration of real-time weather forecasts, larger-scale deployment, and exploration of advanced AI models for further EMS optimization.

## Data Availability

The datasets used and/or analysed during the current study available from the corresponding author on reasonable request.
